# Electrochemotherapy of tumors as in situ vaccination boosted by immunogene electrotransfer

**DOI:** 10.1007/s00262-015-1724-2

**Published:** 2015-06-12

**Authors:** Gregor Sersa, Justin Teissie, Maja Cemazar, Emanuela Signori, Urska Kamensek, Guillermo Marshall, Damijan Miklavcic

**Affiliations:** Department of Experimental Oncology, Institute of Oncology Ljubljana, Zaloska 2, 1000 Ljubljana, Slovenia; IPBS (Institut de Pharmacologie et de Biologie Structurale), CNRS, 205 route de Narbonne BP64182, 31077 Toulouse, France; UPS, IPBS, Université de Toulouse, 31077 Toulouse, France; Faculty of Health Sciences, University of Primorska, Polje 42, 6310 Izola, Slovenia; Laboratory of Molecular Pathology and Experimental Oncology, CNR-Institute of Translational Pharmacology, Via Fosso del Cavaliere 100, 00133 Rome, Italy; Laboratorio de Sistemas Complejos, Departamento de Computación e Instituto de Física del Plasma, CONICET, Facultad de Ciencias Exactas y Naturales, Universidad de Buenos Aires, C1428EGA Buenos Aires, Argentina; Faculty of Electrical Engineering, University of Ljubljana, Trzaska 25, 1000 Ljubljana, Slovenia

**Keywords:** Electroporation, Electrochemotherapy, Gene electrotransfer, Vaccination, Abscopal effect, PIVAC 14

## Abstract

Electroporation is a platform technology for drug and gene delivery. When applied to cell in vitro or tissues in vivo, it leads to an increase in membrane permeability for molecules which otherwise cannot enter the cell (e.g., siRNA, plasmid DNA, and some chemotherapeutic drugs). The therapeutic effectiveness of delivered chemotherapeutics or nucleic acids depends greatly on their successful and efficient delivery to the target tissue. Therefore, the understanding of different principles of drug and gene delivery is necessary and needs to be taken into account according to the specificity of their delivery to tumors and/or normal tissues. Based on the current knowledge, electrochemotherapy (a combination of drug and electric pulses) is used for tumor treatment and has shown great potential. Its local effectiveness is up to 80 % of local tumor control, however, without noticeable effect on metastases. In an attempt to increase systemic antitumor effectiveness of electrochemotherapy, electrotransfer of genes with immunomodulatory effect (immunogene electrotransfer) could be used as adjuvant treatment. Since electrochemotherapy can induce immunogenic cell death, adjuvant immunogene electrotransfer to peritumoral tissue could lead to locoregional effect as well as the abscopal effect on distant untreated metastases. Therefore, we propose a combination of electrochemotherapy with peritumoral IL-12 electrotransfer, as a proof of principle, using electrochemotherapy boosted with immunogene electrotransfer as in situ vaccination for successful tumor treatment.

## Background

It is known that malignant tumors are able to grow and spread because of their ability to escape the immune system surveillance. In fact, there is a process called “cancer immunoediting” that recognizes the existence of a fine interaction between the immune system and tumors, indicating the dual role the immunity plays in cancer [1]. Namely, the immune system not only protects the host against tumor growth, but can promote cancer development by selection of tumor variants with reduced immunogenicity [2]. One promising approach to treat cancer is so-called active immunotherapy, aiming to induce an endogenous tumor-specific immune response in the host. Immunotherapy strategies can include cancer vaccines based on plasmid DNA (pDNA) vectors used to deliver tumor antigens and/or immunomodulatory molecules to stimulate the immune system or oligonucleotides acting on immunosuppressor genes. Current data also support the idea that it is possible to strengthen the anticancer immune response by eliminating or inhibiting the immunosuppressive regulatory T cells (Treg) and by blocking the immune checkpoints [3–5].

In the treatment of tumors, several local therapeutic options are available, from surgery and radiotherapy as prevalent, to thermal ablation techniques, like radiofrequency ablation and cryosurgery, to electrochemotherapy, which is currently being recognized throughout Europe. The effectiveness and safety have brought electrochemotherapy into guidelines for the treatment of different cutaneous and subcutaneous tumors [6]. Recent meta-analysis has evaluated the effectiveness of several ablative skin-directed therapies and clearly indicated the same, or even superior, effectiveness of electrochemotherapy over photodynamic therapy, radiotherapy, intralesional therapy, and topical therapy [7]. Many of these local treatments, like thermal ablation techniques, have different modes of tumor cell death that can elicit different local immune response, which is not sufficient to elicit also strong systemic effect. However, these local treatments could be combined with immune adjuvants that would stimulate a more robust antitumor action and hopefully elicit also a systemic immune response [8]. For some of these local treatments, the elicited immune response resulted in a systemic effect, in so-called abscopal response on distant, nontreated nodules. Such cases were described after radiotherapy [9]. In light of these effects, novel approaches may, when appropriately designed, take advantage of the elicited local immune response and transform it into the systemic response. Electrochemotherapy, a combination of chemotherapy and electroporation, offers itself as another candidate.

In addition to electrochemotherapy, another biomedical application that is based on electroporation is gene electrotransfer, which even though still in early clinical developments has already entered several clinical trials [10]. In the clinical trial with plasmid coding for interleukin 12 (IL-12) electro transferred to some melanoma nodules, the local as well as the loco regional effect on nontreated nodules was observed [11]. Thus, gene electrotransfer of various immunomodulatory molecules could be used for the immunomodulation of the host’s response. In this respect, local ablative effect of electrochemotherapy may set the stage for the enhanced systemic immune response that is elicited by delivery of the therapeutic gene into the organism with immunomodulatory activity.

When using electroporation as a platform technology for drug and gene delivery, electrical parameters must be adjusted for delivery of different molecules and for different target tissues [12, 13]. Electrical parameters that need to be considered are of temporal and spatial nature. Electric pulses that are delivered are of certain duration, shape and amplitude, which characterize their temporal nature. Electric pulses are delivered to cells/tissue via electrodes which, by their shape and positioning, together with the tissue anatomical features and electrical passive properties determine spatial distribution of current density and electric field, and this is to be considered of spatial nature. The community that intends to use this technology needs to be aware of the principles of electroporation effects at the cellular and tissue level based on temporal and spatial consideration, with respect to the specific molecules to be introduced.

Based on the assumption that local treatments can elicit immune response, which can be boosted by gene electrotransfer of immunomodulatory molecules, we need to develop strategies to combine local tumor treatments, such as electrochemotherapy with gene electrotransfer that will be tailored to specific tumor, tissue, and specific mode of action of the therapeutic molecule. In line with this, we describe here, principles of electroporation on cellular and tissue levels. We also propose a strategy, where electrochemotherapy-treated tumor could be used as a live vaccine in conjunction with gene electrotransfer to tumors (intratumorally or peritumorally) boosting local immune response to tumor together with “abscopal effect” on distant metastases.

## Cell electroporation can be controlled

The knowledge of the theoretical background of electroporation is crucial to obtain the most suitable protocol for drug and/or gene delivery [14]. Two key phenomena are induced in the cell membrane: (1) the induced transmembrane voltage, which is crucial for achieving increased membrane permeability, and (2) the transport of the molecules through the permeabilized cell membrane during and after the electric pulse application.

When a cell is exposed to an external electric field *E*, a transmembrane voltage *U*_m_ is induced across the plasma membrane due to the difference between the electric properties of cell membrane, the cytoplasm, and external medium. The induced transmembrane voltage on a spherical cell for a constant electric pulse can be derived from the Laplace equation, which gives a time-dependent solution for the induced transmembrane voltage on a cell membrane [15]. After a membrane capacitance charging time (*t* > 10^−7^ to 10^−6^ s), *U*_m_ can be described as:

1 $$U_{\text{m}} = fg\left( \lambda \right)rE{ \cos }\theta$$

in which *θ* designates the angle between the direction of the normal to the membrane at the considered point on the cell surface and the field direction, *E* the field intensity, *r* the radius of the cell, *g*(*λ*) a function of the specific conductivities of the membrane (*λ*_m_), the pulsing buffer (*λ*_o_) and the cytoplasm (*λ*_i_), the membrane thickness and the cell size and *f*, which is a shape factor (a cell being a spheroid). *U*_m_ is not uniform on the cell surface. These physical predictions were checked experimentally by using potential difference-sensitive fluorescent probes [16, 17]. A key conclusion is that the induced transmembrane voltage depends on the cell size.

When the induced transmembrane voltage exceeds a certain value *U*_c_ (between 0.2 and 1 V), which depends on the pulse parameters—number and duration [18]—the part of the cell membrane where $\left| {U_{\text{m}} } \right| > U_{\text{c}}$ is permeabilized—i.e., electrically conductive defects are formed in the membrane enabling the transport of molecules through the membrane [19].

The molecular reorganization of the membrane associated with this strong increase in transport is not fully understood, but a long-lived alteration of the membrane solution interface was observed [20]. Morphological changes of pulsed cells are present as a long-lived swelling is detected [21] associated with a global change in the membrane rheological properties [22]. No dramatic changes in the membrane organization are present in the long term [23]. However, significant but transient cytoplasmic disorganization occurs. Microtubules and microfilaments are disorganized, while intermediate filaments remain intact [24, 25]. Indeed, these alterations of the cytoskeleton are transient and under strong dependence on the composition of the pulsing buffer. The recovery of the cytoskeleton is associated with the short life of the permeabilized state of the membrane that recovers its selective permeability within a few minutes at physiological temperature [26]. This resealing process is not just due to the viscoelasticity of the membrane, but occurs through defect patching mediated by exocytotic pathways [27].

The membrane structural alterations induced by the electric pulses support transmembrane transport by diffusion of low molecular weight hydrophilic molecules. Membrane permeabilization due to electric pulses is nonselective and molecular flow occurs in both directions. The introduction of drugs like bleomycin or cisplatin is facilitated with consequent increase in their efficiency [12]. An important consequence of increased membrane permeability is also the outflow of secondary metabolites, like adenosine and guanosine triphosphate (ATP, GTP), which affects cell behavior [28, 29]. This may explain in part the observed effects on the cytoskeleton polymerization, which is controlled by these small molecules. Leakage of the ATP is also a danger signal and associated with immunogenic cell death, thus recruiting immune cells. Furthermore, the exposure of cells to electric pulses leads to the formation of reactive oxygen species (ROS) [30], which remain present during the increased membrane permeability.

**Fig. 1:**
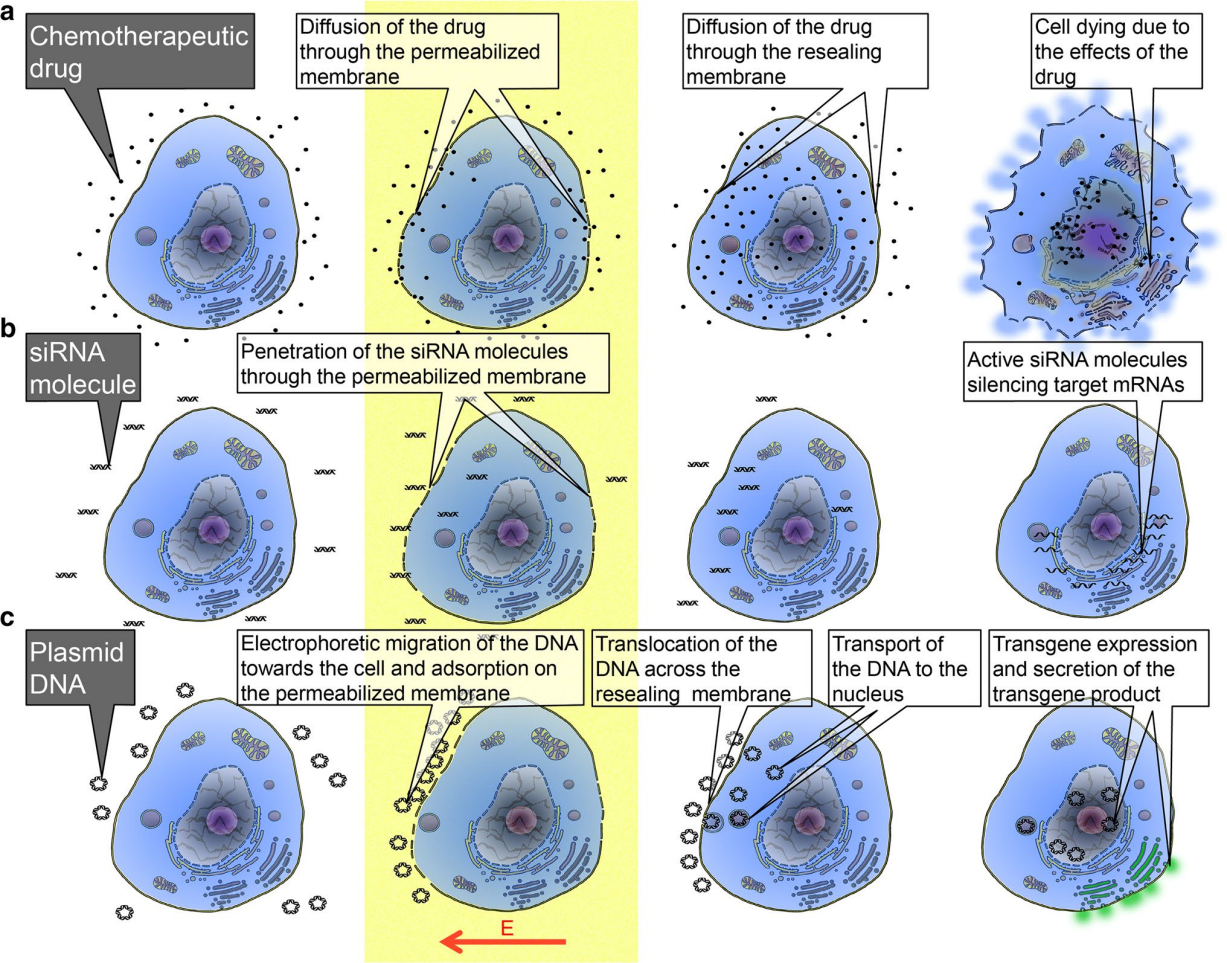
Schematic cartoon showing the processes occurring before, during, and after application of electric pulses (*yellow stripe*) for the delivery of molecules of different sizes into the cell. **a** Delivery of small molecules—an example of electrochemotherapy; **b** delivery of oligonucleotides—an example of siRNA electrotransfer; **c** delivery of larger nucleic acids/macromolecules—an example of pDNA gene electrotransfer

Electrophoretic contribution during the pulse remains negligible in the long-term loading process [31]. Molecular transfer of small molecules (<4 kDa) across the permeabilized area of the membrane is mostly driven by the concentration gradient across the membrane described by the Fick equation during the resealing process, i.e., after the pulse delivery [32] (Fig. 1).

On the contrary, transmembrane translocation of small interfering RNA (siRNA) occurs through the plasma membrane of cells during the electric pulse application [33, 34], which implies that siRNA does not penetrate the cells by diffusion as just described for small molecules. The amount of uploaded siRNA is therefore under the control of the cumulative pulse duration time and the electric field strength (Fig. 1).

In the case of plasmids (pDNA), these macromolecules interact with the plasma membrane by forming long-lived localized aggregates on the electropermeabilized area of the cell membrane [35]. This pDNA/membrane interaction occurs on the side where they are dragged by the electric field-mediated electrophoresis (Fig. 1). Along successive pulses, pDNAs accumulate in a restricted number of aggregates [36] and are translocated across the membrane. Transport within the cytoplasm is another step that happens several minutes after the pulse train. This is an active process under the control of the cytoplasmic ATP level. pDNA can be present in the cytoplasm in a free form that is transported to the nuclear envelope by molecular motors along the microtubules [37] or can be trapped within endocytotic-like actin-covered vesicles [38, 39]. During the residence time of pDNA in the membrane, associated aggregates are affected by the previously mentioned ROS generation. ROS generation can be prevented by adding antioxidants [40]. In summary, the electric pulses induce electrically mediated membrane reorganization, which is a localized event on the cell surface, and occur only when the local field strength is larger than a certain threshold value. Molecules can then cross the membrane giving them access to the cell cytoplasm. Molecular transport through the membrane can thus be controlled by pulse parameters such as amplitude, duration, shape, and number of pulses.

The processes occurring before, during, and after application of electric pulses are schematically presented in the Fig. 1. The delivery of small molecules is shown on an example of electrochemotherapy (Fig. 1a): Before the application of the pulses, the drug is dispersed around the cell; during the pulse and along the resealing of the permeabilized cell membrane, the drug molecules diffuse through the membrane with increased permeability; after the application of the pulses and the membrane resealing, the drug stays trapped in the cell where it can exert its cytotoxic effect. The delivery of oligonucleotides is shown on an example of siRNA electrotransfer (Fig. 1b): Before the application of the pulses, the siRNA molecules are dispersed around the cell; during the pulse delivery, the siRNA molecules translocate through the permeabilized membrane by electrophoresis; after the application of the pulses, the membrane reseals and uploaded siRNA molecules can silence the target mRNAs. The delivery of larger nucleic acids, i.e., macromolecules is shown on an example of pDNA electrotransfer (Fig. 1c): Before the application of the pulses, the pDNA is dispersed around the cell; during the delivery of the pulses, pDNA is dragged by the electric field-associated electrophoresis to the membrane where it adsorbs to the permeabilized part of the membrane and forms localized aggregates; during the resealing of the membrane, the pDNA is translocated across the membrane and transported to the nuclear envelope either by cytoskeletal transport or within endocytotic-like vesicles; once inside the nucleus, the gene encoded by the pDNA is transcribed, and then, therapeutic protein is translated on the ribosomes and, in the case of a gene encoding a secretory protein (for instance IL-12), the product is secreted by the cell; in the case of genes coding for tumor-associated antigens (TAA), proteins will be expressed in their cellular compartments (cell membrane or cytoplasm).

## Drug and gene delivery to cells in tissue depends on additional factors

**Fig. 2:**
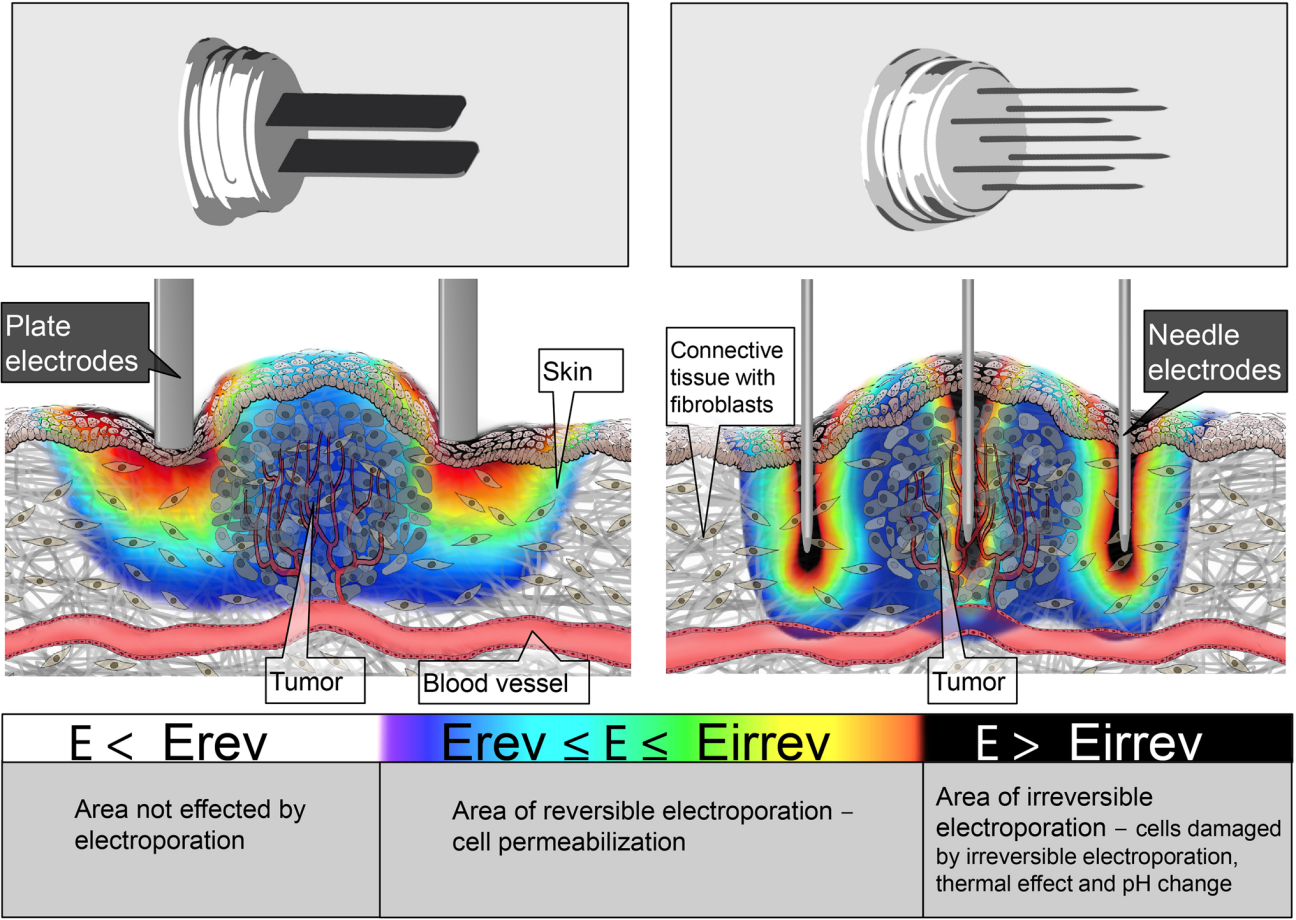
Electric field distribution in and around the tumor tissue during the application of electric pulses for plate (*left*) and hexagonal needle electrodes—cross section through the mid-plane of the electrodes (*right*). The electric field distribution is indicated with the rainbow color scale

The behavior of cells when exposed to electric fields (pulses) cannot be translated directly from cells in vitro to tissues in vivo. Cells in tissue are embedded in a matrix, they are of several types in a certain organ (heterogeneous population), and they form electrical connections between themselves mediated by cell-to-cell junctions [41]. So the values of critical electric field at which cell membrane becomes permeabilized cannot be determined in vitro and then used in in vivo experiments. Furthermore, different tissue properties such as perfusion (better perfusion is usually associated with higher electric conductivity), cell density and cell volume fraction, preferential orientation like in muscle, all affect the electric conductivity of the tissue. Skin has a considerably lower conductivity than any other tissue; muscle conductivity along muscle fibers is higher than in perpendicular direction and tumor tissues generally have higher conductivity than tissue in which they are embedded—even in the liver, which is highly conductive tissue [42]. As a consequence, when we deliver electric pulses using electrodes (either penetrating needles type or nonpenetrating, e.g., surface plate electrodes or pins), the electric current distributes according to the electrode and tissue geometry and tissue conductivity; current and electric field are dual and are connected in place and time through Ohm’s law (Fig. 2). In principle at the same current, higher conductivity will result in lower electric field and vice versa. This means that if electric pulses are applied across the skin, the highest electric field will be in the skin [43]. As electroporation of cell membrane is a consequence of induced transmembrane voltage on the cell membrane [15], cells in high electric field will get permeabilized first. Once they are permeabilized, the conductivity changes and electric field distribution changes as well [44].

It has been shown before that geometry of electrodes and tissue electric properties (i.e., conductivity) determines the electric field which in turn results in increased cell membrane permeability [45]. This means that we can, by choosing different electrodes and controlling their placement with respect to target tissue, achieve controlled membrane permeabilization of target tissues [42, 46]. This is true and well used in treating deep-seated tumors by means of electrochemotherapy [47], but is also true that when using different electrodes in the same in vivo “assay,” different results are achieved [48] as will be discussed later.

Taking into account the nonhomogeneous nature of tissue [43] and that permeabilization of the cell membrane also depends on the duration and number of the applied pulses [18], at the current state of knowledge, it is important to determine experimentally, which is the threshold and necessary electric field to be achieved in target tissue. This was determined to be 400 V/cm for 8 short (100 µs) duration electric pulses, delivered at 1 Hz pulse repetition frequency for the delivery of small molecules to tumors in vivo [49]. The course of tissue permeabilization was studied on a mathematical model of a subcutaneous tumor in small animals [49] and seems also to correspond well in humans based on clinical observations [50]. For rabbit liver using the same parameters, threshold was determined to be 460 V/cm [51, 52]. For rat muscle, the threshold was different depending on whether the pulses were delivered in parallel or perpendicular to the muscle fiber orientation and was 80 and 200 V/cm, respectively [53].

Electric field distribution in and around the tumor tissue during the application of electric pulses for the plate and hexagonal needle electrodes is shown in the Fig. 2. If the electric field is under the threshold for reversible electroporation, the cells are not affected by electroporation (Fig. 2, white areas); if the electric field is above the threshold for reversible and under the threshold for irreversible electroporation, the cells can reseal and survive after electroporation (Fig. 2, blue to red areas); and if the electric field exceeds the threshold for irreversible electroporation, the cells are destroyed/damaged by the effects of irreversible electroporation, thermal effect, and pH changes (Fig. 2, black areas).

For gene electrotransfer in vivo it was shown that membrane permeabilization, which is achieved by high voltage, i.e., permeabilizing pulse(s), is a critical step [54]. But perhaps even more critical is using sufficiently long (from 1 to several 100 ms) low voltage electric pulse or a combination of low voltage electric pulses that are applied after membrane permeabilization, to achieve effective uptake of pDNA into the target cells in tissue [55, 56]. Several different combinations of high voltage, short duration and low voltage, long duration electric pulses were tested and determined empirically for successful gene electrotransfer into different tissues, muscle, skin, tumor, and liver (for specific electrical parameters see [57]). However, several other electrical parameters employing only one type of pulses could also be used for effective gene electrotransfer to tumors muscle and skin [58–61]. Longer pulses, however, were mostly reported to be needed for successful gene transfer.

As the electric field in the tissue depends on tissue properties and anatomy (i.e., geometry), it is important to define target tissue, which is relatively easy in case of tumors and electrochemotherapy, but less in the case of gene electrotransfer aiming at achieving immune response [62]. Depending on the type of therapeutic gene, i.e., tumor antigen or co-stimulatory immune molecules and the target tissues (skin—only dermis or also subcutaneous tissue, muscle, etc.), the target cells in specific tissue are different. Furthermore, it was also shown that target cells are not necessarily present where and when the pulses are applied for the first time [63]. For instance, exposure of skeletal muscle to electric pulses alone causes influx of inflammatory cells that can uptake the pDNA injected several days after the application of electric pulses [63], thus resulting in strong and fast immune response. Furthermore, we need to be aware that for gene electrotransfer, it is mandatory to achieve reversible permeabilization in cells that need to be transfected and avoid irreversible electroporation of these same cells, since they should not be damaged as they need to express the transgene. Finally, we need to be aware that even it might seem rather controversial at the first glance, short membrane permeabilizing, i.e., short, high voltage electric pulses may be less detrimental to the tissue than electrophoretic, i.e., long, low voltage pulses, as they result in higher temperature increase [64], but also in large changes of pH, even extreme changes [65]. However, these are mostly restricted to the immediate vicinity of the electrodes.

Another important aspect that needs to be taken into account when evaluating the gene electrotransfer protocols is the usage of plasmids encoding reporter genes. The results from the studies using reporter genes cannot be directly transferred to the therapeutic application of gene electrotransfer. Different efficiency was achieved by the same electrodes and treatment parameters considering gene expression of reporter gene Luciferase in cells in situ (the ones that were exposed to electric pulses) or at induced immune response of plasmid encoding modified form of human telomerase reverse transcriptase gene (*hTERT*) measured by *hTERT*-specific cytotoxic T cells (CTL) isolated from spleens 14 days after gene electrotransfer to skin [48].

## Electrochemotherapy elicits immune response

Electroporation represents a platform technology for delivery of different molecules to cells and tissues. From its preclinical development, it has quickly been transferred into the treatment of tumors in human and veterinary oncology [13]. Electroporation leads to a transient increase in the permeability of cell membranes when exposed to electric pulses [13]. By use of this technology, specific chemotherapeutic molecules like bleomycin and cisplatin have enhanced uptake in the cells, thus leading to their better cytotoxic effectiveness. This therapeutic approach, electrochemotherapy, has 50–80 % complete response rate of the treated tumors [67], but has only a local effect without systemic effect on distant metastases.

Electrochemotherapy induces apoptotic and necrotic cell death in tumors, and extensive necrotic areas in the tumors are observed after a few days [68]. This leads to tumor antigen shedding in the tumor surrounding. Several lines of evidence support this notion. Recently, properties of immunogenic cell death after electrochemotherapy were demonstrated in a murine tumor model [66].

Firstly, some of the preclinical studies demonstrated infiltration of immune cells into the tumors, indicating an inflammatory reaction [69]. This reaction is due also to the liberation of the cell metabolites that are shed from cells that underwent necrotic cell death, and elicits adaptive immune response [66]. Besides preclinical studies [51], also electrochemotherapy of human melanoma induced maturation of dendritic cells (DC) and their subsequent migration into draining lymph nodes [70]. Furthermore, the release of ATP after electroporation of cells serves also as attractant for DC and their precursors and favors their maturation into antigen presenting cells [66].

Secondly, for complete regression of the tumors after electrochemotherapy, we need to eradicate (kill) all the tumor-initiating (stem) cells. However, due to the technical limits, as well as tissue properties, we cannot effectively cover whole tumor with the sufficient electric field to permeabilize all cells within the tumor, or the drug is not available for the cells’ uptake. Thus, we have to presume that the immune response is responsible for the eradication of all the remaining tumor cells, which is supported by the results of the study where we demonstrated that after electrochemotherapy complete responses of the tumors were obtained in immunocompetent mice, whereas in T cell-deficient nude mice not [51, 71, 72]. Such observations are common also after radiotherapy [73]. Another line of evidence comes from the studies where adjuvant treatment using different cytokines, like IL-2 and TNF-α, were combined with electrochemotherapy resulting in increased antitumor effectiveness [69, 71, 74].

Thirdly, one of the first studies of electrochemotherapy in immunocompetent mice has indicated on the induction of the systemic immune response after electrochemotherapy of tumors. Monocytes isolated from venous blood of electrochemotherapy-treated mice showed increased ability to elicit oxidative burst by production of toxic oxygen species 7 days after treatment. Besides activation of monocytes, which are involved in nonspecific tumor destruction and activation of other components of the immune system, also adaptive immune arm was activated, demonstrated by activation of T lymphocytes. However, this activation may not be sufficient for abscopal effect on distant metastases [75]. On the other hand, none of the in vitro and in vivo studies in mice or clinical studies have demonstrated that electrochemotherapy promotes metastatic process in the organism [75–77]. However, it was shown that electrochemotherapy can induce immunogenic cell death demonstrated by exposure of calreticulin, liberation of ATP, and the release of high mobility group box 1 protein from CT26 colon carcinoma cells in vitro. Such electrochemotherapy-treated cells, when injected into syngeneic mice also protected the animals against tumor challenge demonstrating vaccination effect [66]. Hence, electrochemotherapy may represent an interesting approach to treat solid tumors while preventing recurrence and metastases.

Fourthly, the effect of electrochemotherapy depends also on the immunogenicity of the treated tumors; more immunogenic tumors respond better and with a higher cure rate [78, 79]. In line with these observations, we can also speculate that although electroporation is an effective technology for drug delivery, based mainly on physico-electrical properties, the response rate of the tumors at least to some degree depends also on the tumor type, having in mind also intrinsic sensitivity (resistance) to the chemotherapeutic drug [69, 79, 80]. Some lines of evidence for that exist also for human studies since slight variations in tumor responsiveness were observed in meta-analysis performed on clinical studies on electrochemotherapy published so far [67]. Furthermore, a clinical study on electrochemotherapy of melanoma in patients has demonstrated tumor-infiltrating lymphocytes following treatment, but there was no correlation between their number or distribution and the local response or visceral spread, whereas FoxP3 as a master control gene of Treg was upregulated in tumors that had faster dissemination into the visceral organs [91].

## Electrotransfer of immunomodulatory genes adds a systemic component to electrochemotherapy

Gene electrotransfer is another electroporation-based application, where pDNA or siRNA molecules can be delivered to various tissues, including tumors [81, 82].

Gene electrotransfer is considered to be an effective tool in eliciting antigen-specific immune response in small and large animal models, being responsible for the generation of an inflammatory environment with immune cell infiltration [83]. The migration of these cells seems to be essential to initiate an adequate immune response to the DNA vaccine, proving that this technique is effective in the stimulation of humoral and cellular immunity [84].

Depending on the immunogenicity of the tumors and the immune status of the organism, in vivo gene electrotransfer of pDNAs coding for immunomodulatory molecules such as cytokines, chemokines, adjuvant sequences, siRNA, and/or administration of DNA vaccines carrying tumor-specific or TAA can be used alone or in combination with chemotherapeutics for treatment of tumors [85]. Several therapeutic genes were examined, IL-12, VEGF, PDGF-α, etc. [86–88], but no detailed study of the immune response locally or systemically was conducted. All these studies report that gene electrotransfer is effective in activating the antitumor effectiveness. The therapeutic gene can be administered either intratumorally or peritumorally for predominantly local effectiveness, or into the muscle to induce systemic shedding of the therapeutic molecule. Some studies using IL-2 or IL-12 demonstrated also the antitumor effectiveness on distant untreated tumors and long-term memory of the immune system to tumor cells [69, 89, 90]. As a single treatment, the intratumoral administration of cytokine gene electrotransfer seems the most appropriate way to obtain good therapeutic effect. One of the most studied cytokines is IL-12, with several preclinical studies in different tumor types demonstrating its effectiveness [87], which was proved also in clinical treatment of melanoma patients [86]. Daud et al. [11] have demonstrated that treatment of a few melanoma nodules in the patient can elicit, in certain patients, slow, but an efficient immune response of the organism that results in regression of the treated and also nontreated nodules, as well as prolonged survival without progression of the disease.

**Fig. 3:**
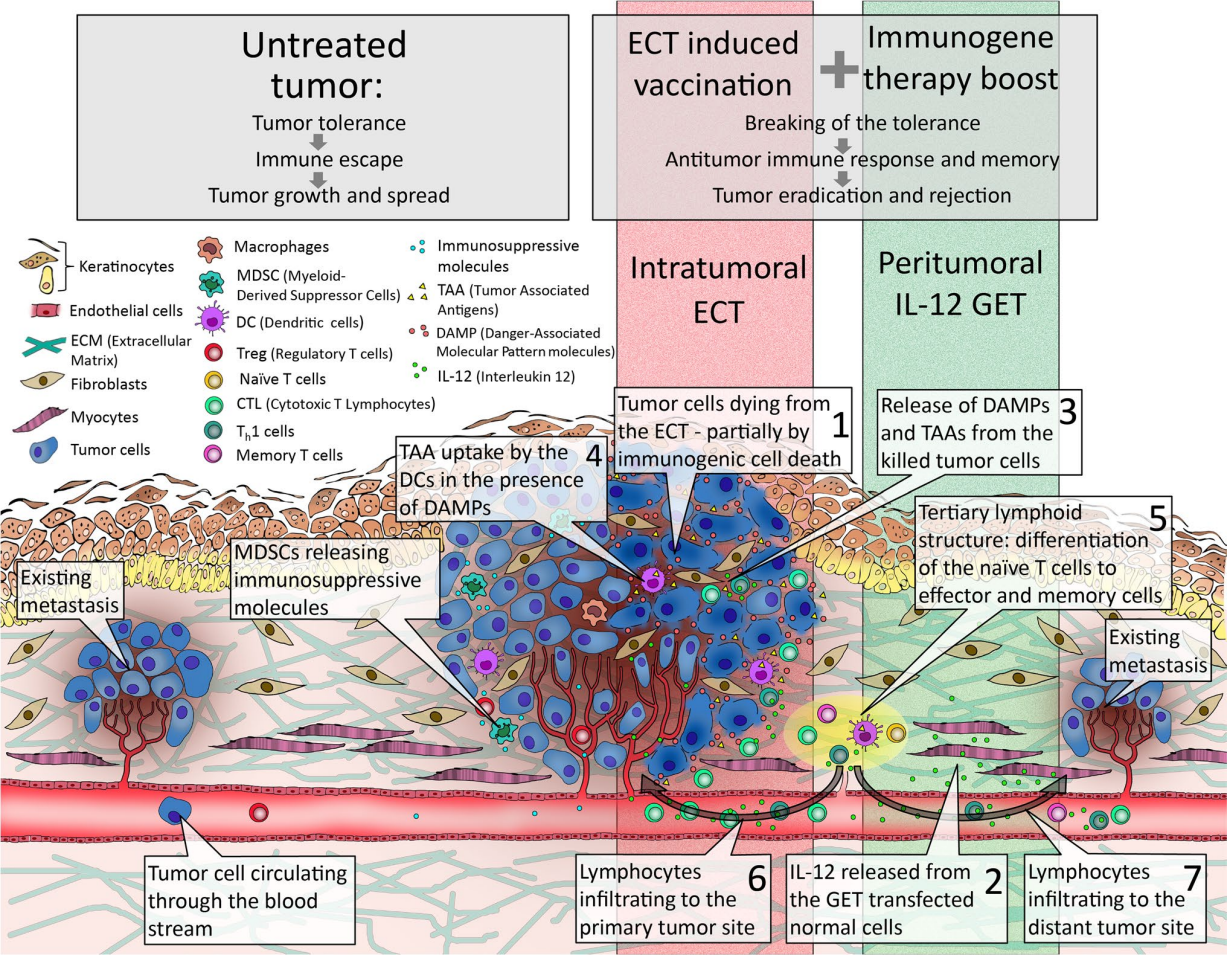
Proposed model for electrochemotherapy of tumors as in situ vaccination boosted by immunogene electrotransfer. *ECT* electrochemotherapy, *GET* gene electrotransfer

Some studies have also explored the combination of local therapies with the gene electrotransfer of immunomodulatory genes. The combinations with either radiotherapy or electrochemotherapy were explored. The strategy was similar, radiosensitization or chemosensitization by immunoadjuvant therapy that boosted the immune response of the organism against tumor [77, 91–96]. A good potentiation of the radiation response and electrochemotherapy was noted, but the underlying immunomodulatory mechanisms were not explored. The knowledge of the mechanisms underlying the elicited immune response by local therapy, such as electrochemotherapy, would lead into the proper scheduling of suitable combination therapies for elicitation of systemic immune response. Thus, appropriate dosage of the pDNA encoding immunomodulatory molecules and the scheduling of it need to be determined [97]. Additionally, suitable biomarkers that would predict the treatment response are needed. Currently, it seems that several consecutive immunogene therapies are necessary. Indeed, in preclinical studies, it was shown that at least three consecutive immunogene electrotransfers should be performed, one prior to electrochemotherapy and two consecutive ones thereafter [78]. Skin gene electrotransfer is very appealing, since the skin is a tissue with vast amounts of immune cells capable of eliciting an efficient vaccination and boosting effect. Some studies have already shown that DC activation in the treatment of melanoma may be an exciting approach [98] (Fig. 3).

The immune response linked to electrochemotherapy and immunomodulatory gene electrotransfer remains to be fully investigated taking into account also the inhibitory function of Tregs, which have been implicated as one of the major suppressive mechanisms of antitumor immune responses. In the tumor environment, Treg-induced immune suppression poses a significant barrier to anticancer responses targeted by immunotherapeutic strategies. Whelan et al. [4] have demonstrated that an increase or decrease in Tregs has a direct influence on the effect of an immunotherapy approach administered by gene electrotransfer of plasmid vectors encoding GM-CSF and B7-1, coupled with the systemic administration of Treg inactivation molecules such as anti-CD25 antibody. More recently, the new results indicate the potential for combining Treg depletion with immunotherapy-based gene electrotransfer into the B16F10 melanoma tumor model to decrease systemic metastasis and potentially enhance survival [99].

Current data also support the idea that it is possible to induce elimination or inhibition of immunosuppressive Treg cells through chemotherapy [100]. Thus, such approach should be especially focused on the possibility to strengthen anticancer immune reactivity. New targeted chemotherapy should therefore be used, preferably for the induction of immunogenic cancer cell death [3].

Here, we propose a model, a combination of electrochemotherapy with immunostimulating peritumoral IL-12 electrotransfer, as a proof of principle that electrochemotherapy can be used as in situ vaccination boosted with immunogene electrotransfer (Fig. 3). In the untreated tumor (Fig. 3, left), immunosuppressive microenvironment with suppressive immune cells like Tregs and myeloid-derived suppressor cells (MDSC) leads to tumor tolerance, and tumor cells escape the immune system and can replicate uncontrollably and spread through the body to form distant secondary tumors. The electrochemotherapy-induced vaccination boosted with IL-12 immunogene therapy (Fig. 3, right) leads to the breaking of the tolerance to otherwise weekly immunogenic intrinsic tumor antigens that result in an antitumor immune response and memory responsible for regression of the treated tumor and untreated distant metastases. Namely, electrochemotherapy-induced tumor cell deaths (Fig. 3, speech balloon 1) combined with IL-12 released into the bloodstream from the transfected cells in the peritumoral region (Fig. 3, speech balloon 2) create a pro-inflammatory microenvironment that leads to recruitment of circulating immune cells. Tumor cells die, at least in part, by immunogenic form of cell death characterized by the shedding of TAA and danger-associated molecular pattern molecules (DAMP) (Fig. 3, speech balloon 3) from the dying cells. Released TAA are captured by DC (Fig. 3, speech balloon 4) that migrate to local lymph node-like structures called tertiary lymphoid structures (TLS) [101] or to the draining lymph nodes (Fig. 3, speech balloon 5) where they initiate adaptive antitumor immune response by priming the naïve T cell to become effector and memory T cells. Tumor-specific lymphocytes, like cytotoxic T lymphocytes and Th1 cells, are then released from the lymphoid structures via circulation and can infiltrate the primary tumor site (Fig. 3, speech balloon 6) and distant metastases (Fig. 3, speech balloon 7) where they exert their immunological actions.

Furthermore, an exciting therapeutic approach to further provide a systemic effectiveness and to enhance the local antitumor response of electrochemotherapy could be the immune checkpoint blockade and/or inhibiting Tregs.

## Conclusion

Membrane electroporation leading to increased membrane permeability is a phenomenon which allows the introduction of non- or poorly permeant molecules into the cells. The key factors governing electroporation are the amplitude of induced transmembrane voltage which depends on the electric field to which the cell is locally exposed, to the cell size, shape, and its orientation in the field. It is important to stress that the local electric field *E* is the critical parameter for membrane electroporation/permeabilization as it defines the area of the membrane which is permeabilized and through which ionic and molecular transport occurs [102].

Similar to the effects at the molecular and cellular level, a considerable amount of knowledge has been accumulated about properties of tissues and how these affect cell electroporation in the tissue and consequently drug and gene electrotransfer. In spite of the fact that the exact mechanisms involved at the molecular level of the cells and tissue electroporation are not fully understood, it is possible to determine a set of electrical parameters, providing safe and efficient procedure for in vivo applications that could be translated into clinical use.

Electrochemotherapy is an efficient local ablative treatment, which is currently employed in numerous oncology centers throughout Europe. However, it is a local treatment, which would need a systemic component that would boost the immune response of electrochemotherapy itself. Therefore, gene electrotransfer of immunomodulatory molecules in the peritumoral skin could add this systemic component, by enhancing locoregional and/or systemic response. Hence, we propose a strategy, where electrochemotherapy-treated tumor could be used as a live vaccine in conjunction with gene electrotransfer to tumors.

## Abbreviations

ATP: Adenosine triphosphate
B7-1: B7 protein
CTL: Cytotoxic T cells
DAMP: Danger-associated molecular pattern molecules
DC: Dendritic cells
E: Electric field
GM-CSF: Granulocyte-macrophage colony-stimulating factor
GTP: Guanosine triphosphate
hTERT: Human telomerase reverse transcriptase
IL-12: Interleukin 12
IL-2: Interleukin 2
PDGF-α: Platelet-derived growth factor
pDNA: Plasmid DNA
ROS: Reactive oxygen species
siRNA: Small interfering RNA
TAA: Tumor-associated antigens
TLS: Tertiary lymphoid structures
TNF-α: Tumor necrosis factor alpha
Treg: T regulatory cells
*U*_c_: Critical voltage
*U*_m_: Transmembrane voltage
VEGF: Vascular endothelial growth factor
